# The microstratigraphy and depositional environments of Lida Ajer and Ngalau Gupin, two fossil-bearing tropical limestone caves of west Sumatra

**DOI:** 10.1038/s41598-023-50975-8

**Published:** 2024-01-02

**Authors:** Holly E. Anderson, Mike W. Morley, Conor McAdams, Jahdi Zaim, Yan Rizal, Aswan, Mika R. Puspaningrum, Agus T. Hascaryo, Gilbert J. Price, Julien Louys

**Affiliations:** 1 Australian Research Centre for Human Evolution, Environmental Futures Research Institute, Griffith University, Brisbane, QLD 4111 Australia; 2 Archaeology, College of Humanities, Arts and Social Sciences, Flinders University, Adelaide, SA Australia; 3 Geology Study Program, Institut Teknologi Bandung, Bandung, Jawa Barat 40132 Indonesia; 4 School of Earth and Environmental Sciences, The University of Queensland, Brisbane, QLD 4072 Australia

**Keywords:** Evolution, Solid Earth sciences

## Abstract

Lida Ajer and Ngalau Gupin are karstic caves situated in the Padang Highlands, western Sumatra, Indonesia. Lida Ajer is best known for yielding fossil evidence that places the arrival of *Homo sapiens* in Southeast Asia during Marine Isotope Stage 4, one of the earliest records for the region. Ngalau Gupin recently produced the first record of hippopotamid *Hexaprotodon* on the island, representing the only globally extinct taxon in Pleistocene deposits from Sumatra. Microstratigraphic (micromorphological) analyses were applied to unconsolidated fossil-bearing cave sediments from these two sites. We use micromorphology as part of a micro-contextualised taphonomic approach to identify the diagenetic processes affecting fossils and sediments within these caves, through phases of their depositional history. The fossil-bearing sediments in Lida Ajer have been subjected to a suite of natural sedimentation processes ranging from water action to carnivore occupation, which would indicate the fossils underwent significant reworking prior to lithification of the deposit. The results demonstrate that the base of the unconsolidated fossil-bearing sediments in Ngalau Gupin were derived from the interior of the cave, where the matrix was partially phosphatized as a result of guano-driven diagenesis. These observations can be used to test hypotheses about the integrity of incorporated vertebrate remains and to aid in local palaeoenvironmental reconstructions. The methods employed in this research have not previously been applied to cave sediments from sites in the Padang Highlands and provide key new insights into the palaeontological and natural history of the western region of Sumatra.

## Introduction

Discoveries of fossil *Homo sapiens* in Southeast Asia are most frequently made in cave deposits (e.g. Demeter et al.^1^; Westaway et al.^2^; Freidline et al.^3^). Excavated by Eugène Dubois in the late nineteenth century, the cave deposits in the Padang Highlands of west Sumatra have been used as evidence for the early presence of modern humans in Southeast Asia (Westaway et al.^2^; Louys et al.^4^). However, recent research has highlighted the significant risk of time- or habitat-averaging due to natural ‘mixing’ processes of remains in complex cave environments (e.g. O’Connor et al.^5^; Duringer et al.^6^; Louys et al.^7^; Smith et al.^8^). Mixing can lead to inaccuracies in establishing the age of deposits, as fossils that have very different taphonomic histories may become interred in a single lithostratigraphic layer. While sophisticated microstratigraphic methodologies have been developing over the past few decades, they are rarely applied in a Southeast Asian setting (Morley & Goldberg^9^; Smith et al.^8^; Smith et al.^10^; Smith et al.^11^). The lack of detailed knowledge about the taphonomic context, the history and development of Southeast Asian cave fossil assemblages, and about time, depth, and deposition of the fossil-bearing sediments in these sites hinders the establishment of reliable links with dating efforts (Louys et al.^7^; Smith et al.^8^; Smith et al.^10^; Smith et al.^11^). This has important implications for interpreting the date of early human arrival and other faunal occurrences in the region.

Recent palaeontological research in the Padang Highlands has generated significant taphonomic data important in establishing the stratigraphic provenance and temporal positions of fossil-bearing deposits in tropical caves (e.g. Louys et al.^4^^,^^7^; Westaway et al.^2^; Duval et al.^12^; Smith et al.^10^; Smith et al.^11^). Smith et al.^8^ demonstrated that the focus of taphonomic research in Southeast Asia has steadily changed to the geomorphological aspects of cave formation, though successful micromorphology applications to determine the agents of concentration that have influenced the cave sedimentary deposits and incorporated faunal assemblages in the region are still rare. Exceptions include micromorphological work at Niah cave, Sarawak (Stephens et al.^13^^,^^14^), Batadomba Lena cave (Kourampas^15^), Liang Bua cave, Flores (Morley et al.^16^), and Con Moong cave, Vietnam (McAdams et al.^17^^,^^18^). Data generated from micromorphological analyses in Southeast Asia provide detailed information regarding the fundamental mechanisms of accumulation of tropical cave sediments and any inclusions, including organic components such as vertebrate remains. This method originated in soil science and became widely used in archaeological science (Goldberg^19^^–^^21^; Goldberg & Berna^22^; Karkanas & Goldberg^23^^–^^25^; Shahack-Gross et al.^26^^,^^27^), documenting formation processes on a microscopic level using techniques from petrographic microscopy to more sophisticated methodologies such as Fourier Transform Infrared Spectroscopy (FTIR) and Scanning Electron Microscope (SEM) (e.g. Morley et al.^16^; McAdams et al.^17^).

**Figure 1:**
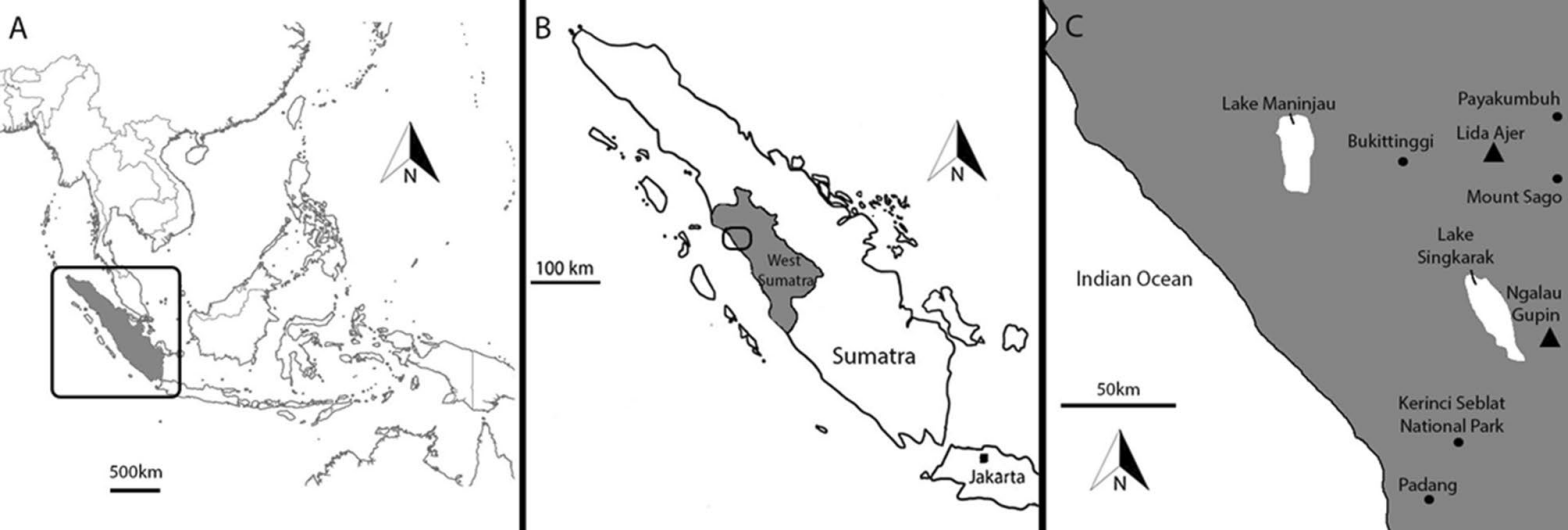
(**A**) Left. A map of Southeast Asia with a box highlighting the location of Sumatra; (**B**) Map of Sumatra with shading highlighting the location of west Sumatra and a box highlighting the region in which Lida Ajer and Ngalau Gupin are situated; (**C**) map of the region of west Sumatra, with Lida Ajer and Ngalau Gupin indicated (Triangles). Figure created using Adobe Inc. (2019). Adobe Photoshop 2024. Retrieved from https://www.adobe.com/products/photoshop.html.

During a survey of caves situated in the Carboniferous-Permian limestone hills of the Padang Highlands of western Sumatra, two key localities, Lida Ajer and Ngalau Gupin, were chosen for analysis and excavation due to a considerable presence of undisturbed fossil-bearing sediment deposits. The human and non-hominin fossils recovered from Ngalau Gupin and Lida Ajer (Fig. 1), reveal the sole presence of *Hexaprotodon* in Sumatra and one of the earliest records of modern humans in Southeast Asia, respectively (Smith et al.^10^, Westaway et al.^2^; Louys et al.^4^). There is significant potential for the use of micromorphology at these sites to explore the taphonomy of the assemblages and reveal the diagenetic histories of the fossiliferous deposits. These data may permit future researchers to identify and restrict the ecological reliability of the faunas preserved at these sites for the reconstruction of paleoenvironments. One of our primary concerns is to preserve the original integrity of the cave deposits and to analyse the depositional relationships that might otherwise have been lost while employing conventional (bulk) sampling methods. Here, we report the micromorphological results for one undisturbed sediment block from Ngalau Gupin and three undisturbed sediment blocks from Lida Ajer.

## Cave morphometry and sediments

Lida Ajer and Ngalau Gupin caves are formed in Carboniferous-Permian limestones as part of extensive karst systems and are currently infilled with karstic breccia and unconsolidated sediments. The sediments deposited in these sites are primarily diamicts, comprising sandy clays, muds and silts with mammal teeth incorporated in some of these fine-grained deposits. Recent excavations of the fossil-bearing chambers in these caves have uncovered the complexity of the karst deposits within, revealing detailed sedimentology histories and fossil assemblages produced by carnivores but subsequently accumulated by porcupines (Westaway et al.^2^; Louys et al.^4^^,^^7^; Smith et al.^10^).

### Lida Ajer

**Figure 2:**
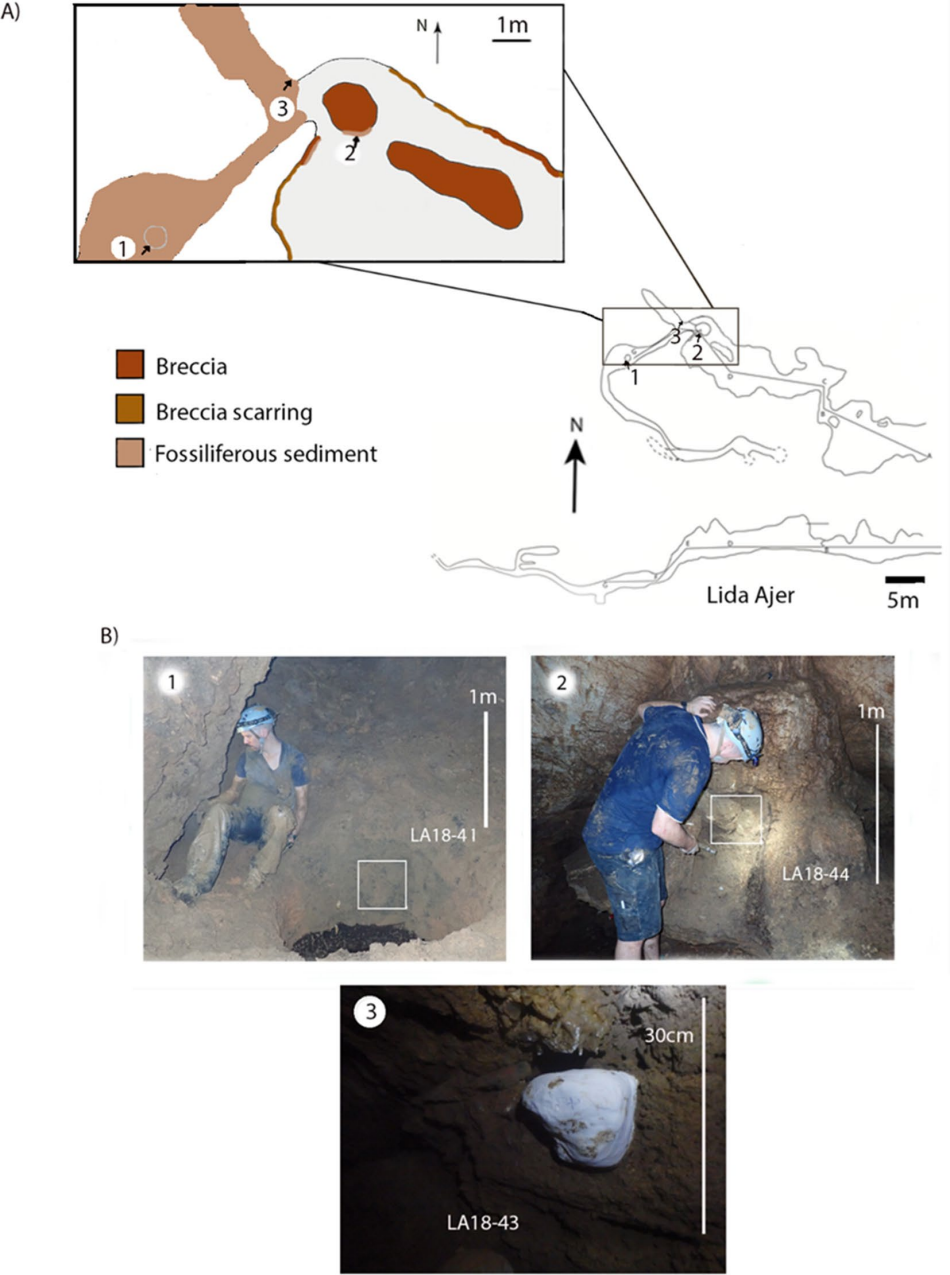
(**A**) scale profile map of Lida Ajer. Arrow indicates the orientation of the photographs in (**B**) in relation to the inset scale profile map (above) and a scale plan map of Lida Ajer (inset) with a colour scale section highlighting the key breccia and fossiliferous sediment sites; (**B**) Photograph of the key fossiliferous sediment sites in Lida Ajer, the white border highlighting the exact extraction point of the micromorphology samples. LA18-41 is at or near the interface between stratigraphic units 4 and 5a, LA18-44 is unit 7, and LA18-43 is unit 5a, as described in Louys et al.^4^. Figure created using Adobe Inc. (2019). Adobe Photoshop 2024. Retrieved from https://www.adobe.com/products/photoshop.html.

The Lida Ajer cave entrance is 4.8 m wide and 2.1 m high, and the interior comprises three main chambers and a rear sinkhole that extends below the first fossil-bearing chamber (Fig. 2A). The cave contains unconsolidated fossiliferous sediments located in two main areas. The first is visible on the walls and cave floor in the northern corner of the first fossil-bearing chamber. The sediments have a surface area of approximately 1.5 × 7 m (Fig. 2). The second is through the sink hole. The fossiliferous sediments are visible on the walls and floors of the sink hole passageway from the entrance to termination. The sediments cover a surface area of approximately 30 × 5 m and extend to approximately 1.5 m (see Louys et al.^4^: Fig. 2).

### Ngalau Gupin

**Figure 3:**
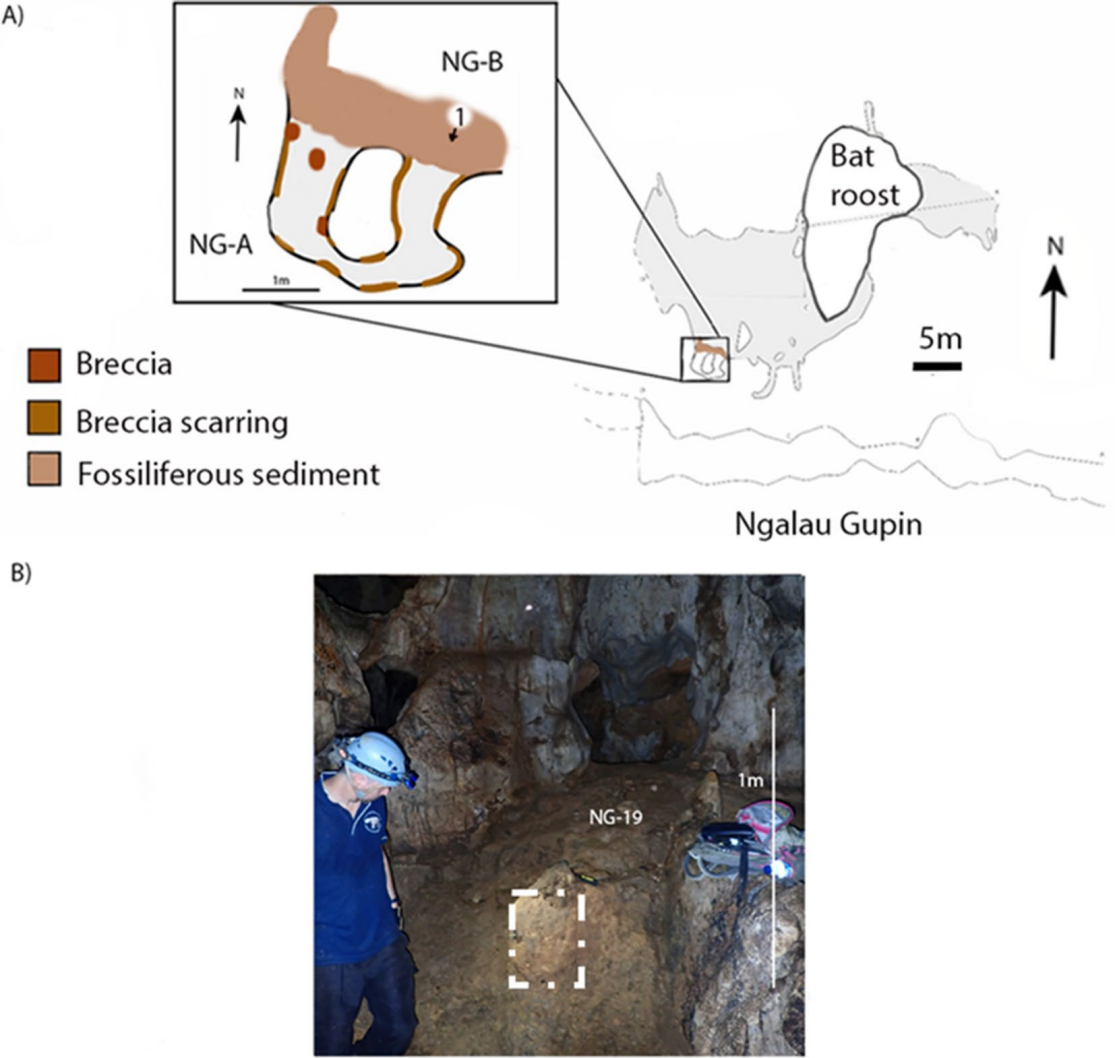
(**A**) scale profile of Ngalau Gupin. Arrow indicates the placement and orientation of the photograph in (**B**) in relation to the inset scale profile map (above) and a scale plan map of Ngalau Gupin (inset) with a colour scale section highlighting the key breccia and fossiliferous sediment site; (**B**) Photograph of the key fossiliferous sediment sites in Ngalau Gupin, the white border highlighting the exact extraction point of the micromorphology sample. Figure created using Adobe Inc. (2019). Adobe Photoshop 2024. Retrieved from https://www.adobe.com/products/photoshop.html.

The Ngalau Gupin cave entrance is 12 m wide and 5 m high, and the interior comprises one main chamber with a U-shaped passageway in the south-east corner (Fig. 2C) preserving fossils. Ngalau Gupin also has unconsolidated fossil-bearing sediments visible on the topmost layer of the floor, directly outside of the U-shaped passageway in the southern extent of the main chamber (Fig. 3A). The unconsolidated sediments have a main surface area of approximately 5 by 5 m, with a section of one-by-one metres stretching from the west passage at a depth of approximately ten centimetres, and visibly erodes to muddy topsoil (Fig. 3B).

## Site backgrounds

De Vos^28^ described over 10,000 fossils excavated from Lida Ajer cave by Dubois and documented an assemblage predominantly comprised of porcupine-gnawed tooth crowns which represent a rich and diverse Late Pleistocene fauna (Proboscidea, Primates, Artiodactyla, Perissodactyla, Carnivora) largely analogous to extant fauna in the modern rainforests of Sumatra. Hooijer^29^ described two human teeth amongst the mammalian assemblage excavated from Lida Ajer cave. At the time these results were published, the location of the Lida Ajer cave site remained unvisited by western scientists since the original excavations by Eugene Dubois. There were uncertainties about the age of the breccia in which the hominin remains were found, as direct dating analyses were not possible and thus the results lacked a robust chronology. A morphological analysis of these teeth by Westaway et al.^2^ determines that the combination of the small size and external and internal morphology demonstrates that they derive from anatomically modern *Homo sapiens*. Thus, Westaway et al.^2^ returned to Lida Ajer cave and performed a multi-disciplinary, direct dating study to constrain the age of the breccia deposits within. The depositional model from these data suggested the breccia formed between 73 and 63 kyr in a single depositional event by fluidised mass movement, which would indicate the fossils underwent only minor reworking prior to lithification of the deposit. These hypotheses have recently been updated, however, and Louys et al.^4^ suggests that sediment deposition during MIS 4 infilled the sinkhole passages and lower main fossil chamber of Lida Ajer with fossil-rich muds under alternating high and low energy flow conditions. Louys et al.^4^ considered that the human presence represented by the dental remains (as well as all other mammalian fossils) were likely deposited during MIS 4, though recommended further direct dating to determine the exact units these early humans were recovered from.A palaeontological analysis of the vertebrate remains within Ngalau Gupin was recently undertaken by Smith et al.^8^. The remains were excavated from two loci within the cave site, named NG-A and NG-B; the former comprising the cemented karst breccia on the cave walls and the latter consisting of the fossiliferous sediments covering the cave floor immediately below NG-A. Abundant fossils were excavated from the breccias cemented on the cave walls and floors of Ngalau Gupin, consisting of mostly isolated teeth from small-to-large-sized animals. The collection in Smith et al.^10^ reveals a rich, diverse Pleistocene faunal assemblage (Proboscidea, Primates, Rodentia, Artiodactyla, Perissodactyla, Carnivora) largely analogous to extant fauna in the modern rainforests of Sumatra. The only exception is the hippopotamid *Hexaprotodon*, which represents the only globally extinct taxon in deposits from Sumatra and the first record of this animal from the island. Analysis of the taphonomic and taxonomic data derived from the vertebrate remains suggests the assemblage originated as a prey accumulation from a large carnivore outside of the cave, which was then gnawed upon by porcupines within the cave. The study establishes a depositional model linking the formation of the NG-A and NG-B sites. Smith et al.^8^ suggested that, following modification by porcupines, the remains were cemented into the NG-A assemblage to form a consolidated breccia. Erosion and decalcification of the deposit led to the remains falling from the NG-A breccia, to be redeposited onto the top of the unconsolidated NG-B sediments directly below, aided by low energy water flow resulting in minor lateral and vertical movement before final burial.

Ngalau Gupin and Lida Ajer allow us to compare the fossil formation processes operating at two sites from the same region, that formed at around the same time, which, superficially at least, resemble one another in terms of their sediment fills. The microstratigraphic complexity of the deposits complicates the interpretation of formation, preservation, and destruction of these sites, which is a critical factor in understanding the palaeontological and palaeoenvironmental history of the region.

## Methodology

To carry out the microstratigraphic analyses, intact blocks of oriented sediment (~ 15 × 15 cm) were extracted from each profile face in gypsum plaster jackets to retain integrity. Once extracted and returned to the Flinders University Microarchaeology Laboratory, these samples were unwrapped and oven-dried at 35 °C. Each block was impregnated with a clear polyester resin, diluted with a styrene monomer at a ratio of 7:3 and catalysed with methyl ethyl ketone peroxide (MEKP). Once cured, the resinated sediment was cut into 75 × 50 mm 'wafers’ of 1 cm thickness with a circular saw fitted with a diamond encrusted masonry saw blade. These wafers were cut and polished down to 35 μm and mounted on glass slides by Adelaide Petrographics. Thin-sections were observed using a polarising microscope at magnifications ranging from 8 × to 200 × under plane-polarised light (PPL) and cross polarised light (XPL). Twelve thin sections were cut from the unconsolidated sediments in total: three from LA18-44 – Lida Ajer Unit 7 of Louys et al.^4^; three from LA18-43 – Unit 5a from the right passage of the sinkhole of Lida Ajer; three from LA18-41 – the top of the sinkhole pit, at or just above the boundary between Units 4 and 5a of Louys et al.^4^; and three from NG19—the unconsolidated sediment immediately below the fossil-bearing NG-B of Ngalau Gupin. Each thin section was given a unique number (Table 1). Thin section terminology follows that of Stoops^30^.

**Table 1:** Sample ID, site and locality of the twelve thin sections from Lida Ajer and Ngalau Gupin.

Sample ID	Site	Locality
LA18-41 (1)	Lida Ajer	Top of sinkhole pit/the interface between units 4 and 5a
LA18-41 (2)	Lida Ajer	Top of sinkhole pit/the interface between units 4 and 5a
LA18-41 (3)	Lida Ajer	Top of sinkhole pit/the interface between units 4 and 5a
LA18-43 (1)	Lida Ajer	Right passage of sinkhole/unit 5a
LA18-43 (2)	Lida Ajer	Right passage of sinkhole/unit 5a
LA18-43 (3)	Lida Ajer	Right passage of sinkhole/unit 5a
LA18-44 (1)	Lida Ajer	Breccia site 2–fossil chamber/unit 7
LA18-44 (2)	Lida Ajer	Breccia site 2–fossil chamber/unit 7
LA18-44 (3)	Lida Ajer	Breccia site 2–fossil chamber/unit 7
LA18-44 (4)	Lida Ajer	Breccia site 2–fossil chamber/unit 7
NG-19 (1)	Ngalau Gupin	Main chamber NG-B
NG-19 (2)	Ngalau Gupin	Main chamber NG-B
NG19 (3)	Ngalau Gupin	Main chamber NG-B

## Microstratigraphy results

### Lida Ajer

#### Sediment block sample LA18-41 – base of unit 5a

**Figure 4:**
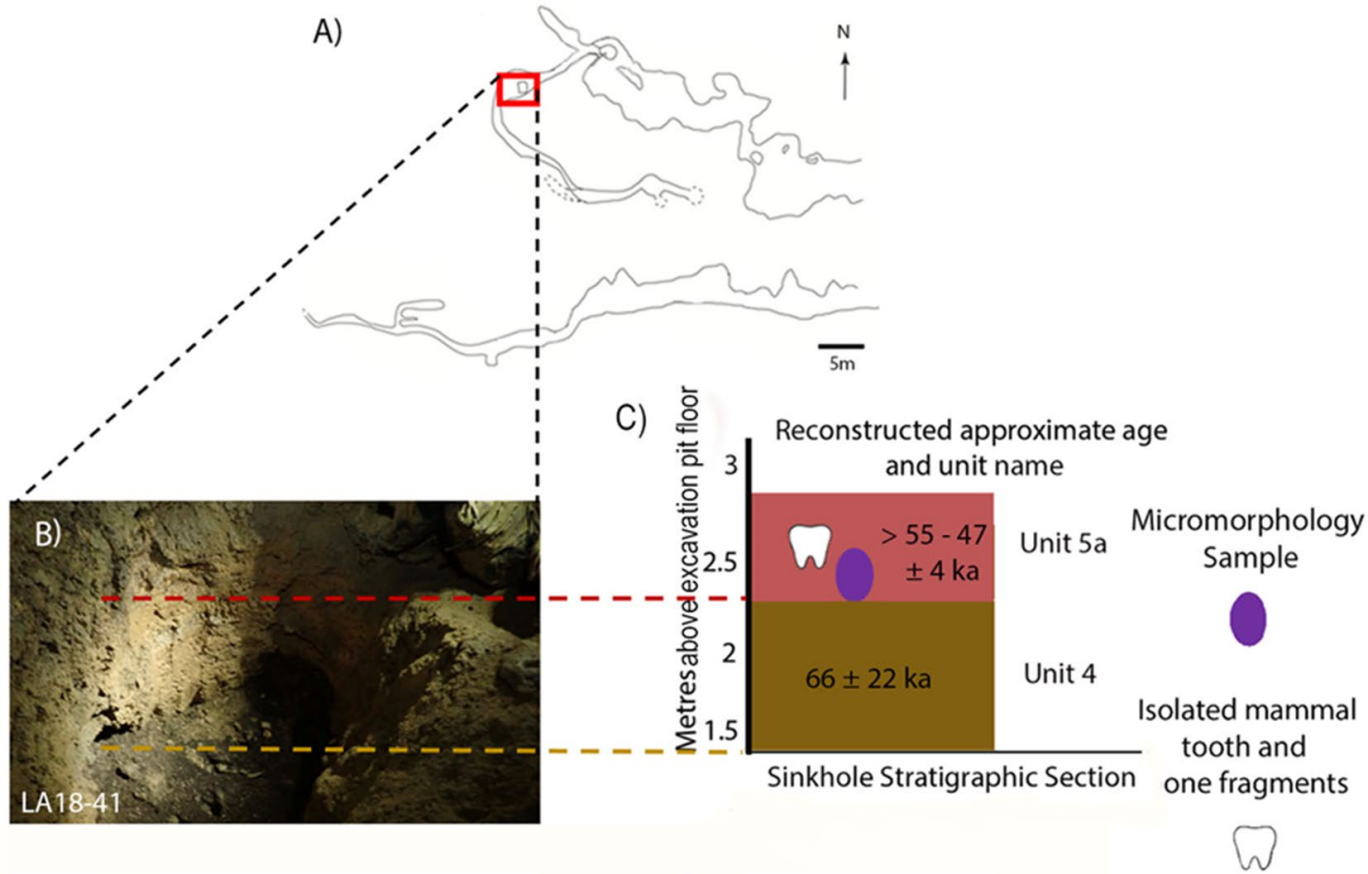
(**A**) (Above) Scale profile and (below) scale plan of Lida Ajer. Red box denotes location of the excavation pit (**B**) Photograph of Lida Ajer excavation pit in which micromorphology sample LA18-41 was taken; (**C**) Stratigraphic section of sedimentary units 4 and 5a in Lida Ajer excavation pit as surveyed in Louys et al.^4^. Units numbered in reference to stratigraphy as denoted in Louys et al. (2022). Figure created using Adobe Inc. (2019). Adobe Photoshop 2024. Retrieved from https://www.adobe.com/products/photoshop.html.

**Figure 5:**
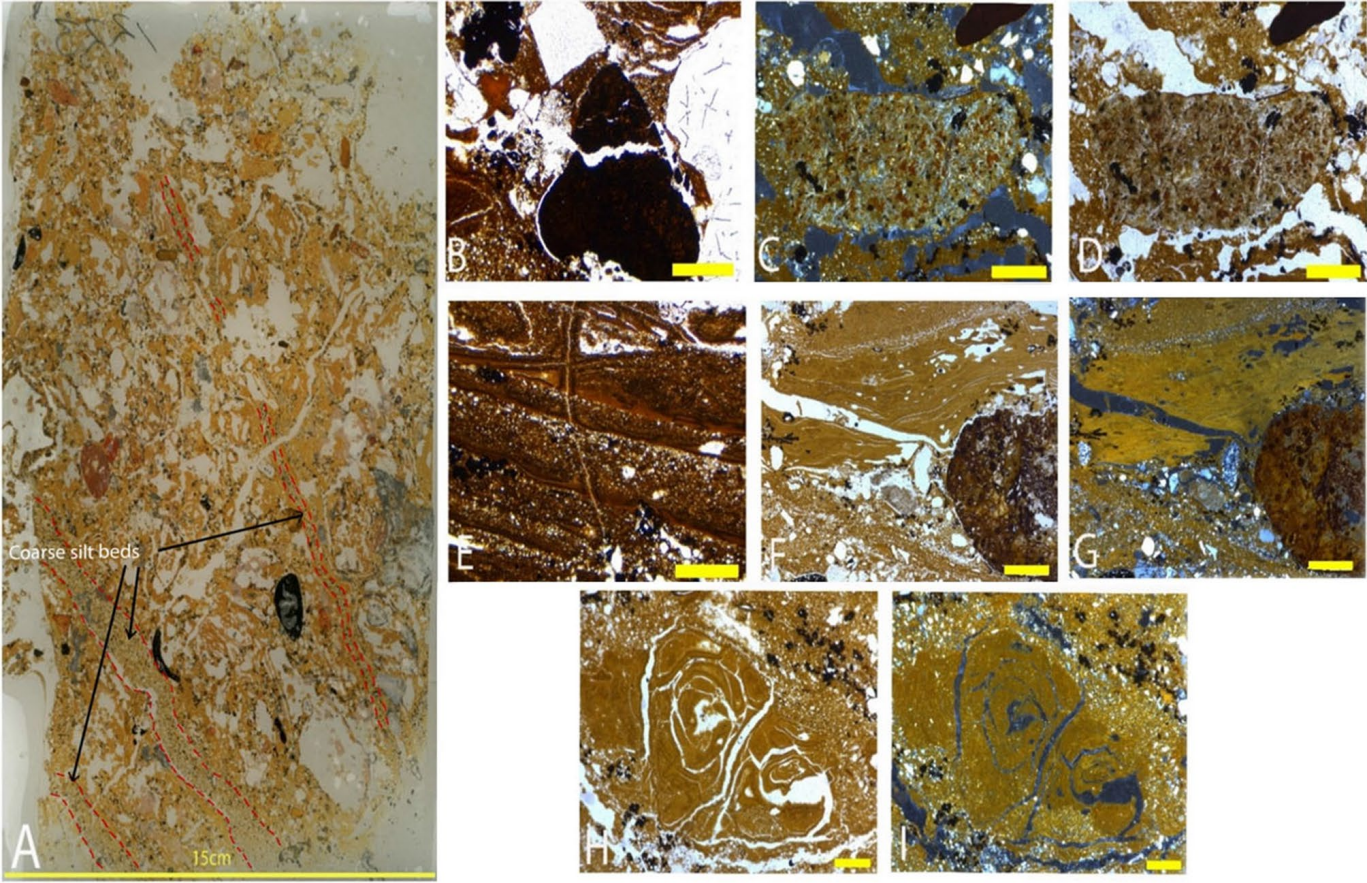
(**A**) Thin section LA18-41; Red dashed lines denote sharp contacts between basal diamict and sharp silt beds, the latter highlighted by black arrows: 2.5 × magnification photomicrographs: (**B**) Fractured rip up clast with large mineral grains, and clay and silt infilling voids in PPL; (**C**) Large, rounded compound aggregate with clays and acicular crystal formation in the clays in PPL; (**D**) and in XPL; (**E**) Silt and clay laminations in PPL; (**F**) Blocks of laminated clay that may be in-situ with sand lenses beneath, and phosphatic nodule to right of the image in PPL; (**G**) and in XPL; (**H**) Clay infill which is cracking in situ, and Mn staining in PPL; (**I**) and in XPL. Figure (**B**–**I**) Yellow bar indicates 1 mm in size.

The location of sediment block LA18-41 was chosen to target a sediment exposure created by an excavation pit most likely dug under supervision by Dubois (Price et al.^31^) and resolve the transition between barren and fossil-bearing sediments (Fig. 4). The thin sections are described below, from the lowest to highest in the sedimentological sequence (Fig. 5A). Dating of sediment using OSL techniques has returned ages of 66 ± 22 ka from Unit 4, while isolated teeth from Unit 5 has provided ages of > 55 ka and > 47 ± 4 ka (Louys et al. in 2022^4^).

##### Thin section LA18-41 (1)

Thin-section LA18-41(1) displays a well-sorted texture overall, comprising a clay-rich basal diamict divided from several coarse stratified silt beds by sharp contacts (See Fig. 5A). Grading upwards in this diamict the sediment is an increasingly chaotic mix. The base of the sediment sequence in this slide is comprised of laminations of dusty clays and fine silt infilling inclined at 30 degrees and interbedded with evaporites (See Fig. 5F,G). Clay infills are visibly shrunk and cracked (See Fig. 5H,I). Within the basal diamict is a heterogeneous array of well-rounded discoidal and irregular clasts, which are stained dull black or red with a unimodal orientation. Rounded orange-yellow stained bone fragments are randomly dispersed throughout the matrix. The old organics that are present in this sample are heavily iron stained and humified. Ubiquitous manganese staining is evident throughout these laminations.

##### Thin section LA18-41 (2)

The sediments in this thin-section are chaotic, dense and mechanically fractured, associated with rip-up clasts and with no bedding visible (See Fig. 5E). There is an upward-fining distribution of banded silts to fine clay (See Fig. 5G,H), though the sediments are heavily reworked and the spongey aggregates intermix with the grainier sediment. Incorporated clasts range in size from 0.4 to 2.5 cm in size. Figure 5I is formed from varied rock types, including angular quartz grains, speleothem fragments, metamorphic quartzite, and quartz sand. Clastic material displays a horizontal orientation. Black, orange and red clasts are heavily fractured and randomly dispersed throughout the matrix; fibrous organic material is visible within the clay matrix and the peripheries of lighter-coloured clasts. A large brown clast in the sample appears to have several clasts incorporated within, a dark brown vein structure running through it and a pitted texture. Very rounded bone fragments are evident throughout the whole matrix, with some yellow-orange staining. Banding is seen at the base of the sequence, though not in-situ, and manganese and iron are present throughout the sample.

##### Thin section LA18-41 (3)

Throughout this sample are very calcareous sediments, which are heavily bioturbated and weathered. Laminated silt and clay layers, up to 1 mm thick are visible in the lower portion of the sample (Fig. 5E–G). Translocated clays form a fractured coating over all aggregates in the matrix, including several sparse fragments of chert and a large singular speleothem fragment. Clay infills all the voids in the sample (Fig. 5B–D). Burrowing of insects is evident in the clays (See Fig. 5H,I), however, no fossil remains are visible in this sample.

#### Sediment block LA18-43 – top of unit 5a

**Figure 6:**
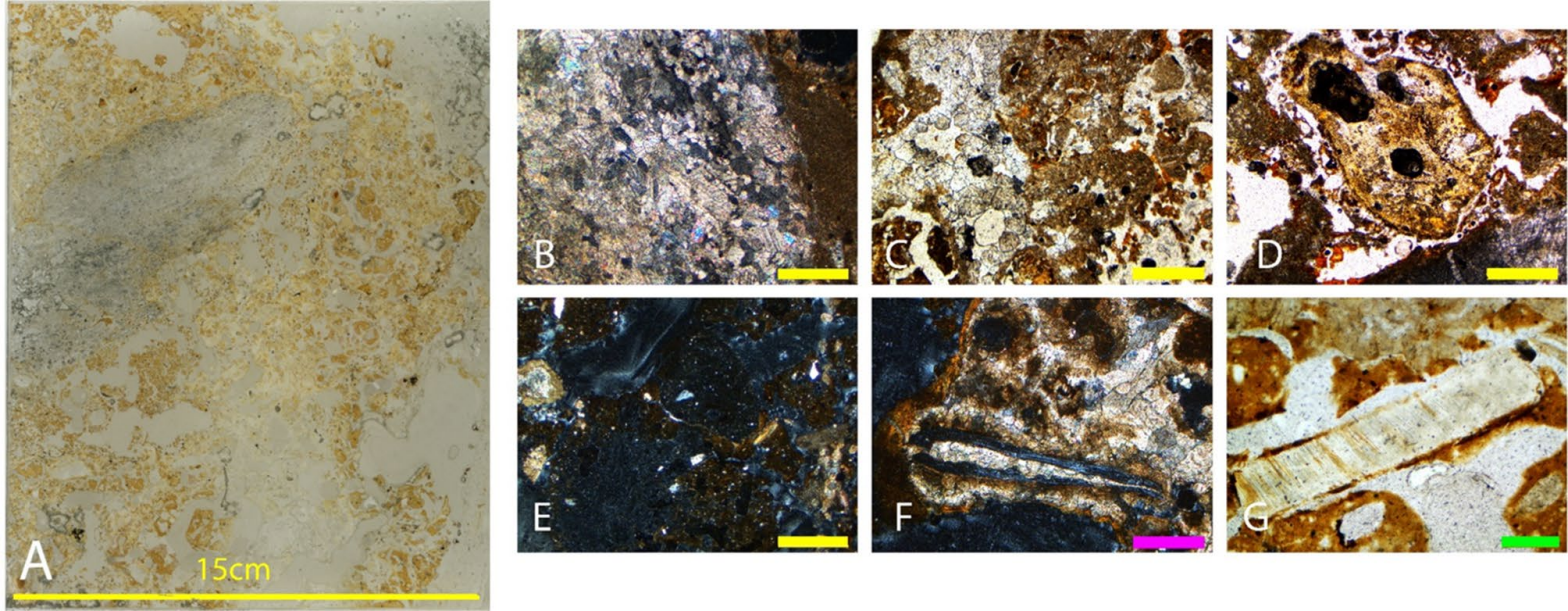
(**A**) Thin section LA18-43; 2.5 × magnification photomicrographs: (**B**) Matrix with calcium carbonate in the void spaces in XPL; (**C**) Carbonate sand break down of speleothem in phosphatic environment in PPL; (**D**) Coprolite fragment in PPL; (**E**) Coprolite fragment in phosphatic matrix in XPL; (**F**) Bone fragment in calcite sand matrix with clay in XPL: 10 × magnification; (**G**) Shell fragment in clay coating in PPL. Figure (**B**–**G**) Yellow bar indicates 1 mm in size, pink bar indicates 200 μm in size and green bar indicates 500 μm in size.

The location of sediment block LA18-43 was chosen to further resolve the depositional history of the sediments in Unit 5a. The sediment from the wall of the sinkhole passage is correlated with Unit 5b, and the block under examination is in close proximity to the sinkhole entrance (Louys et al.^4^). The thin sections from this sediment block are described below, from the lowest to highest in the sedimentological sequence (See Fig. 6A).

##### Thin section LA18-43 (1)

There is not a lot of variation throughout the sequence in this sample, with the sediment separated into just two distinct fabrics comprising a pale-yellow spongey matrix chaotically mixed with dark orange-brown silt/clay. Broken clay coatings are visible and voids are variously infilled with reprecipitated calcite or silty clay infills. These silty clays contain many clasts of variable size, angularity, and colour; the most abundant of the clastic aggregates is chert, which is sparsely interspersed throughout the sediments and coated with clay. There are several yellow stained rounded bone fragments averaging one centimetre in size and speleothem fragments within the matrix.

##### Thin section LA18-43 (2)

This is a much more clay-rich sequence than the previous slide and broken up clay is also intermixed with the sediments. Calcite has precipitated in voids throughout the sequence, particularly at the top of the sequence where there is more open porous sediment (See Fig. 6B,C). At the base of the sequence, there is a clay coating that infills the void spaces with apparent weathering. There are clastic aggregates, some of which are also coated in clay. Dusty yellow brown coprolites are evident throughout the sequence, containing blackened organic material, highly degraded bone fragments, hair clumps, phytoliths, egg shell and a coarse fraction dominated by chert fragments and quartz grain (Fig. 6E–G). The coprolites have a distinct outer surface separate from the surrounding sediment, though are internally fractured. Most of the specimens have typical ‘faecal’ sub rounded morphology, though several are irregular. There are only two fossils in the sample, a thin needle of bone evident at the top of the sequence (See Fig. 6H) and a singular tooth, which notably has preserved organics. There is a large piece of flowstone apparent at the base of the sequence, with a dark rim around it (Fig. 6D).

##### Thin section LA18-43 (3)

This slide is primarily comprised of phosphatised flowstone overlain by clay-rich calcitic sediments. There are dark areas of intense calcification, calcite overprinting and breaking up of older calcite with a decreasing abundance of clay grading upwards through the sample (Fig. 6B,C). Flowstone is apparent with partially decalcified interlocking crystals. There are several coprolites (Fig. 6D,E), as well as bone fragments (Fig. 6F) that have become spongey, compressed, and broken in the upper section of the sequence, and shell fragments (Fig. 6G). A singular very large speleothem fragment is evident in the matrix.

#### Sediment block LA18-44 – unit 7

**Figure 7:**
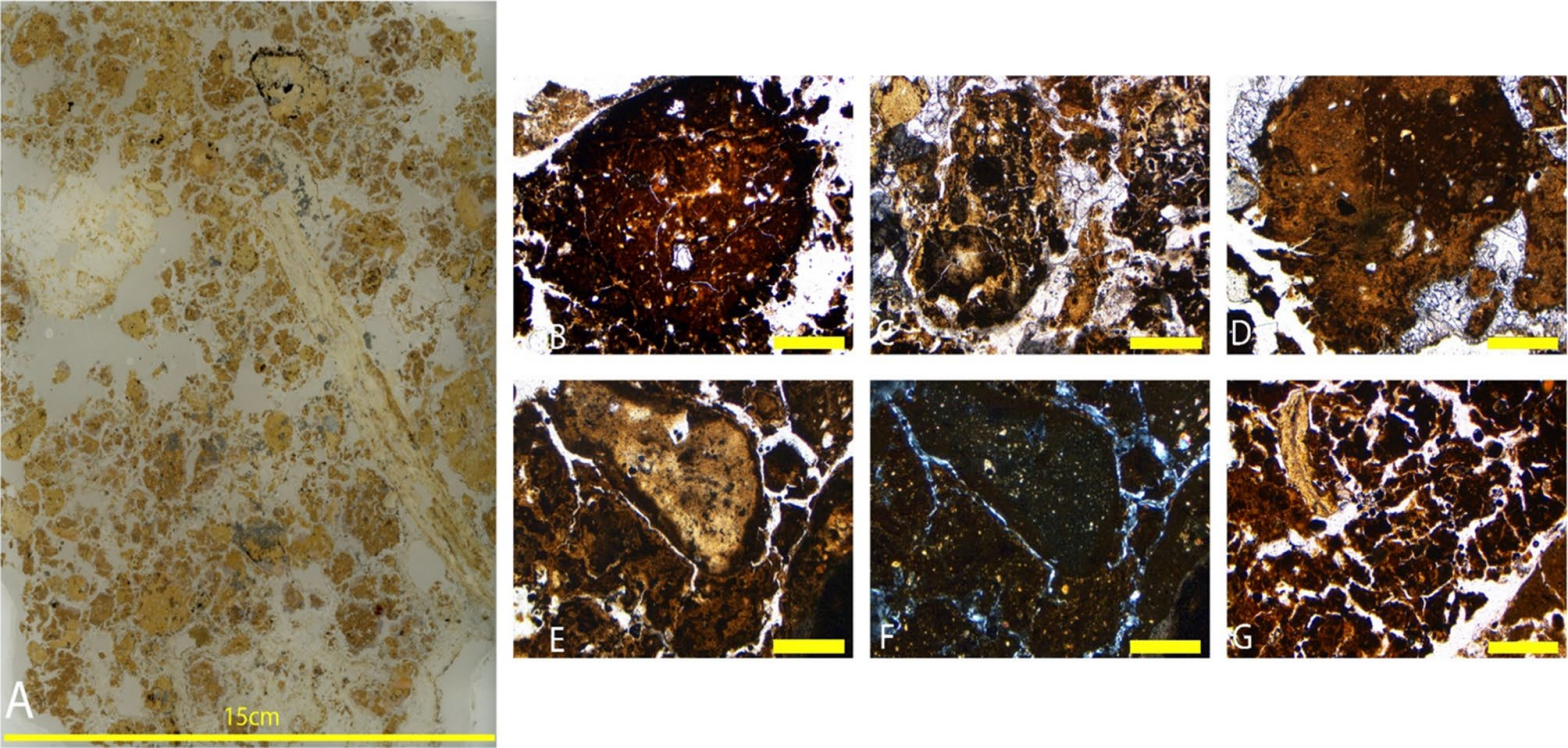
(**A**) Thin section LA18-44; 2.5 × magnification photomicrographs: (**B**) Coprolite fragment containing course fraction and bone fragments in PPL; (**C**) Degrading coprolite fragment in PPL; (**D**) Large coprolite in calcite sand in PPL; (**E**) Coprolite in phosphatic matrix in PPL; (**F**) and in PPL; (**G**) Bone in heavily bioturbated matrix in PPL. Figure (**B**–**F**) Yellow bar indicates 1 mm in size.

The location of sediment block LA18-44 was selected to resolve the depositional history of the sediments that infilled the main fossil chamber of Lida Ajer. The block sample is from the north-facing profile exposed in the entrance of the cave sinkhole. Dating of breccia sediments, fossil teeth and calcite at site two of the fossil chamber constrains the age of deposition at 68 ± 5 ka (Westaway et al.^2^). The thin sections from this sediment block are described below, from the lowest to highest in the sedimentological sequence (See Fig. 7A).

##### Thin section LA18-44 (1)

This sample is dominated by calcareous silts with only a small proportion of translocated finer material. Void spaces are entirely infilled by quartz grains between 0.1 and 0.3 mm in size or precipitation of calcite and phosphatic diagenesis affects the matric across much of the sample. Translocation of the silts in the groundmass into void spaces is apparent throughout the sample as the matrix is very broken up, and lots of cracking is seen in the sediments in-situ. The whole sample contains abundant dusty yellow brown coprolites with a high frequency of blackened organic material, bone fragments, hair clumps, phytoliths and a coarse fraction dominated by chert fragments and quartz grains (Fig. 5B). Numerous coprolites are internally fractured though not displaced (Fig. 5C).

##### Thin section LA18-44 (2)

This slide is comprised of rip-up clasts suspended in a disturbed clay-silt matrix that has abundant void spaces, associated with secondary carbonates. Quartz, silt or fine sand are visible within the coprolites and there is frequent weathered coprolitic material throughout the sample (Fig. 6D–F). Amorphous phosphatic nodules are associated with the coprolites and notably, there is carbonate crystallisation. Calcite in the deposit is micro-stratified and post-depositionally neo-formed into the void spaces, which appears like a geode with a growth ring evident. There is a singular speleothem fragment ~ 2–3 cm in size at the top of the sequence.

##### Thin section LA18-44 (3)

This sample is comprised of a silt matrix with a small amount of incorporated clay and little movement of the finer material in the sample. Amorphous phosphates are visible at the base of the sample and, grading upwards, the sediments are increasingly broken up and mechanically disturbed. Void spaces are visible throughout, in which precipitation of calcite is common. There are numerous aggregate grains throughout the sample, namely rip-up clasts in a disturbed clay-silt sequence, and there are coprolites throughout approximately 60% of the sample (Fig. 7B–F). The coprolites are heavily broken up, both in-situ and ex-situ, and are generally welded together with carbonate crystals. There are also quartz, silt, and fine sands in the coprolites, the latter two of which have yellowed due to phosphates. There is significant bioturbation in 80% of the sample, small bone fragments (Fig. 7G), and burrowing is evident throughout the matrix.

### Ngalau Gupin

#### Sediment block NG-19 – base of NG-B sediments

**Figure 8:**
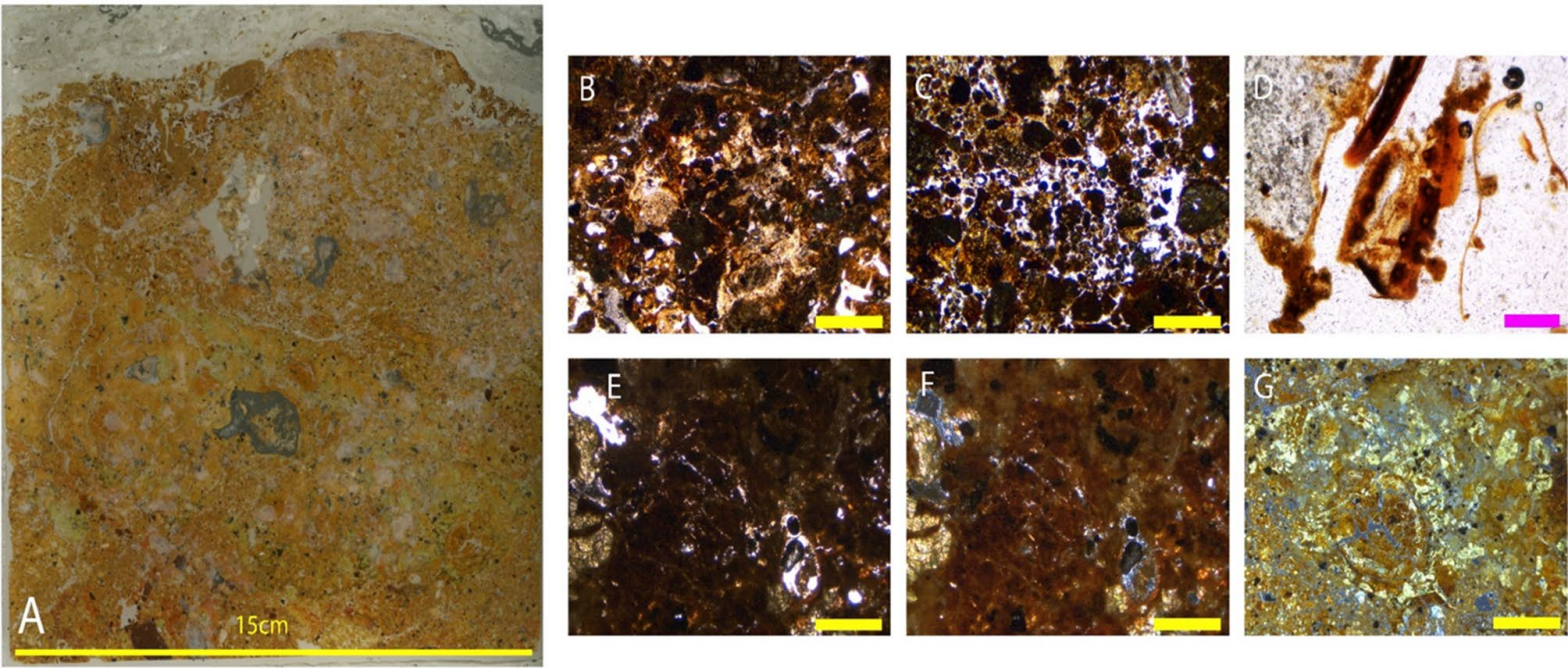
(**A**) Thin section NG19 (1); 2.5 × magnification: (**B**) Phosphatic deposition in the matrix in PPL; (**C**) and in XPL; (**D**) Chitinous remains; (**E**) Phosphatic groundmass; (**F**) Degradation of abundant guano deposits in PPL; (**G**) and in XPL. Figure (**B**–(**G**) Yellow line indicates 1 mm in size and pink line indicates 200 μm in size.

**Figure 9:**
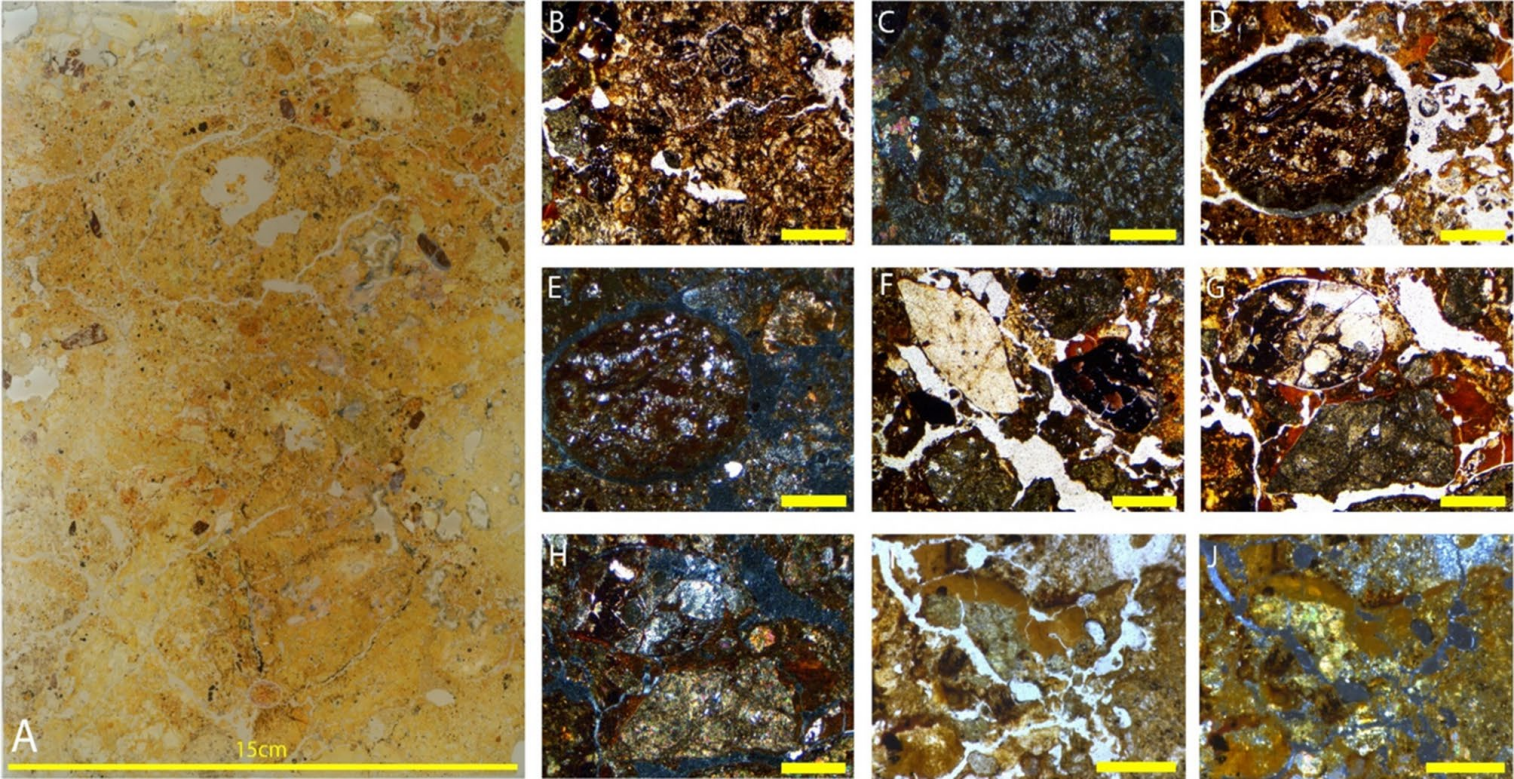
(**A**) Thin section NG19 (2); 2.5 × magnification: (**B**) Phosphatised groundmass in PPL; (**C**) and in XPL; (**D**) Phosphatic nodule in PPL; (**E**) and in XPL; (**F**) Varied clasts of different origins in PPL; (**G**) Granostriated clay halo around varied clasts in PPL; (**H**) and in XPL; (**I**) Welded clays with Mn and very phosphate-rich environment including weathered phosphates (**J**) and in XPL. Figure (**B**–**J**) Yellow bar indicates 1 mm in size.

**Figure 10:**
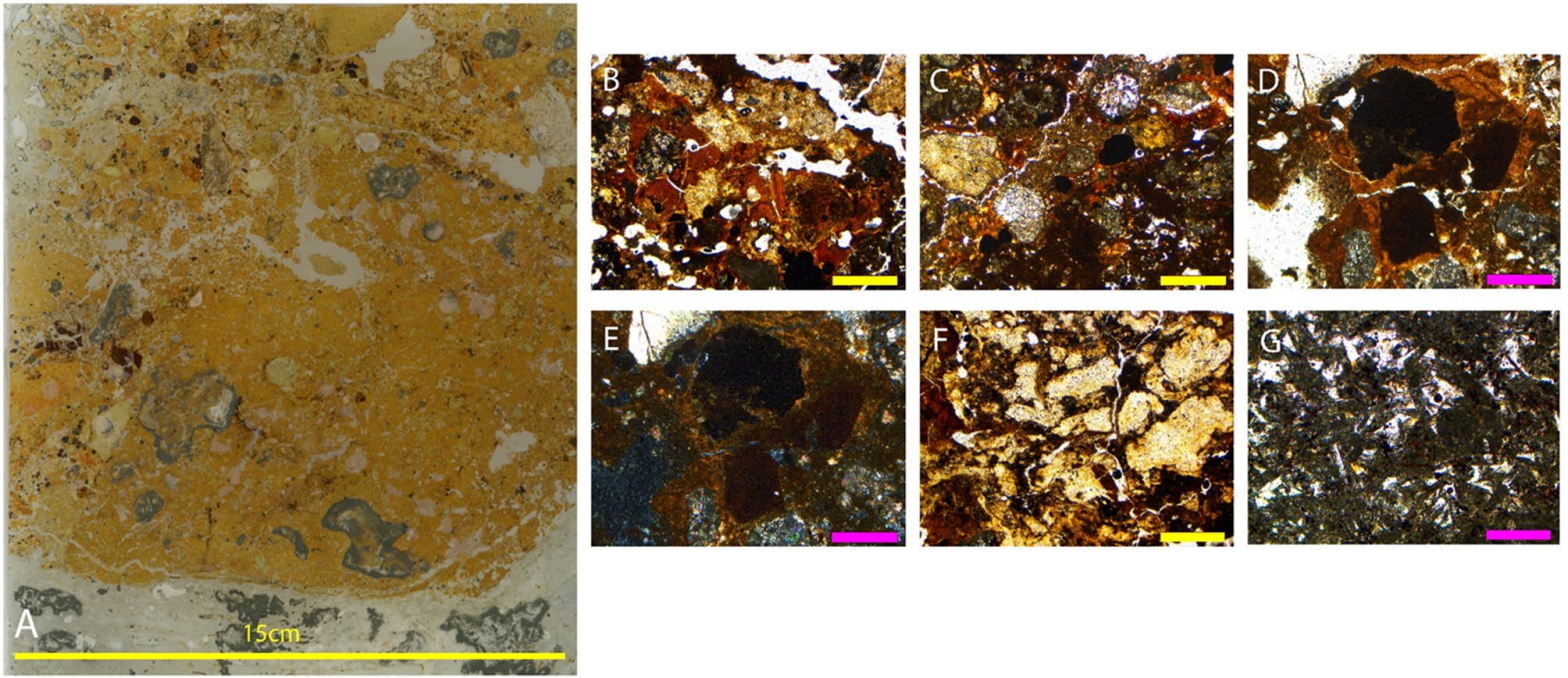
(**A**) Thin section NG19 (3); 2.5 × magnification: (**B**) Abundant clay deposits in PPL; (**C**) Clay infilling void space in PPL; 5 × magnification: (**D**) Granostriated clays surrounding clasts in PPL; (**E**) in XPL; 2.5 × magnification: (**F**) Highly phosphatic deposits derived from guano; 5 × magnification: (**G**) Gypsum fragments with lenticular crystals in PPL. Figure (**B**–**G**) Yellow line indicates 1 mm in size and green line indicates 500 μm in size.

The location of sediment block NG-19 was chosen to assess whether there is any stratigraphy to be resolved in the unconsolidated deposits below the fossil-bearing sediments. The block sample is from the deepest sediment deposit with the aim of preserving as undisturbed and detailed a profile as possible. The geological age of the fossil remains in the Ngalau Gupin deposit based on the teeth preserved in NG-A and NG-B, assuming the depositional model is correct, are approximately 160–115 ka, following the US-ESR results presented in Smith et al.^10^. The thin sections from this sediment block are described below, from the lowest to highest in the sedimentological sequence (See Figs. 8A, 9A and 10A).

##### Thin section NG-19 (1)

This slide is a very heterogeneous mix of carbonates and clays (Fig. 8A), potentially including a range of authigenic phosphate minerals (Fig. 8B,C). Towards the base of the section, there are contiguous layers of birefringent clay infills that have weathered and broken up (Fig. 8G) and a lot of aggregates incorporated into the matrix including gypsum fragments, decomposed plant materials and plant pseudomorphs (Fig. 8B & C). The sediment appears organic, with broken down chitinous insect remains dispersed throughout the matrix (Fig. 8D). There is black staining of groundmass and abundant guano deposits throughout the sample (Fig. 8E,F) with restricted areas of very bioturbated sediments and infilled burrows at the scale of 200 to 500 μm (Fig. 8F,G).

##### Sample NG-19 (2)

This sequence is a dirty mixture of fine silt sediments that are phosphatised to some extent (Fig. 9B–E). Towards the top of the sequence, this opens to a more porous sediment which shows significant weathering of pore spaces infilled with clays. Common in the groundmass is a mealy-coloured clay, which has optical properties that are suggestive of phosphate minerals under crossed polarised light (XPL). Lots of clay and gypsum aggregates are present, with clay-rich welding of clasts liberated from older clays and heavily weathered phosphates. Banding and orientation of the clasts becomes apparent within the centre of the sample, though there remains dusty clay domains which are all broken up and undifferentiated. Fresh clays become increasingly chemically altered as the matrix becomes phosphate rich and there is phosphate weathering locally (Fig. 9I,J). There are several different fine-grained rock types randomly distributed throughout the chaotic mix of sediments (Fig. 9F). There is a halo around the clay clasts (Fig. 9G & H) and those clasts which are fragmented in the matrix have very weathered, partially fractured crusts.

##### Sample NG-19 (3)

This slide sample, and the entirety of the Ngalau Gupin sample, has much more dense and impermeable sediments than those described above from Lida Ajer; comprising a pure grey colluvial deposit with abundant clays (Fig. 10B & C). The abundance of clay increases up sequence and the orientation of the clay particles in the groundmass leads to a granostriated fabric (Fig. 10D & E), which creates birefringence under cross polarised light. Lots of clay, bat guano and gypsum aggregates are present in this layer, and phosphate minerals are forming (Fig. 10F & G).

## Discussion

Micromorphological analysis of the Lida Ajer and Ngalau Gupin deposits has revealed micro-contextual features that are indicative of site depositional environments. These features are the result of destructive processes altering tropical sediments and ultimately destroying the incorporated skeletal remains in caves around the world, though conflictingly and contrastingly act as key evidence to reconstruct knowledge of site formation processes and ancient palaeoenvironmental conditions. These features provide an opportunity to reconstruct the interrelated taphonomic histories of incorporated faunal remains within. This micromorphological dataset improves upon previous interpretations based on taxonomic and taphonomic analyses and field observations, enhancing our understanding of the temporal reconstruction of local palaeoenvironment and regional chronostratigraphies in the Padang Highlands of Sumatra.

### Hominin activity in Lida Ajer & Ngalau Gupin caves microstratigraphic record

Two isolated human teeth – a molar and a premolar – identified by Hooijer, remain the sole indication of hominins at Lida Ajer (Westaway et al.^2^). There is no evidence to date of a hominin presence in Ngalau Gupin (Louys et al.^7^; Smith et al.^10^). There were no macroscopic signs of hominin activity, such as combustion structures or artefacts (e.g. Aldeias et al.^32^; Mallol et al.^33^), observed during field study and this micromorphological investigation has not revealed any evidence for hominin occupation in Lida Ajer and Ngalau Gupin caves, though it must be considered that this outcome could be due to sampling bias, given the limited spatial area encompassed by our study. Smith^34^ determined that while it may be plausible that the presence of carnivorous coprolites in Lida Ajer could be to some extent attributed to hominins, this cannot be confirmed as it is not possible to ascertain specific producers or occupation events. Hominins and carnivores do not cohabitate (Villa et al.^35^; Morley et al.^36^) and determining whether there has been alternating hominin–carnivore occupations at Lida Ajer would require further research. There is no direct evidence that carnivores may be responsible for the human remains found in the Lida Ajer cave, though this scenario is certainly plausible.

### Carnivore occupation events at Lida Ajer cave microstratigraphic record

An important outcome of our study is the identification of microstratigraphic features consistent with carnivore occupation, namely abundant coprolites. Dedicated middens and latrines marked by ubiquitous faecal deposits are direct indication of frequent dwellings in caves (e.g. Carrión et al.^37^; Dean^38^; Reinhard et al.^39^; Taru & Backwell^40^, Morley et al.^36^). The abundant coprolite record in Lida Ajer cave suggests the site was used by animals for prolonged intervals of the site depositional histories.

**Figure 11:**
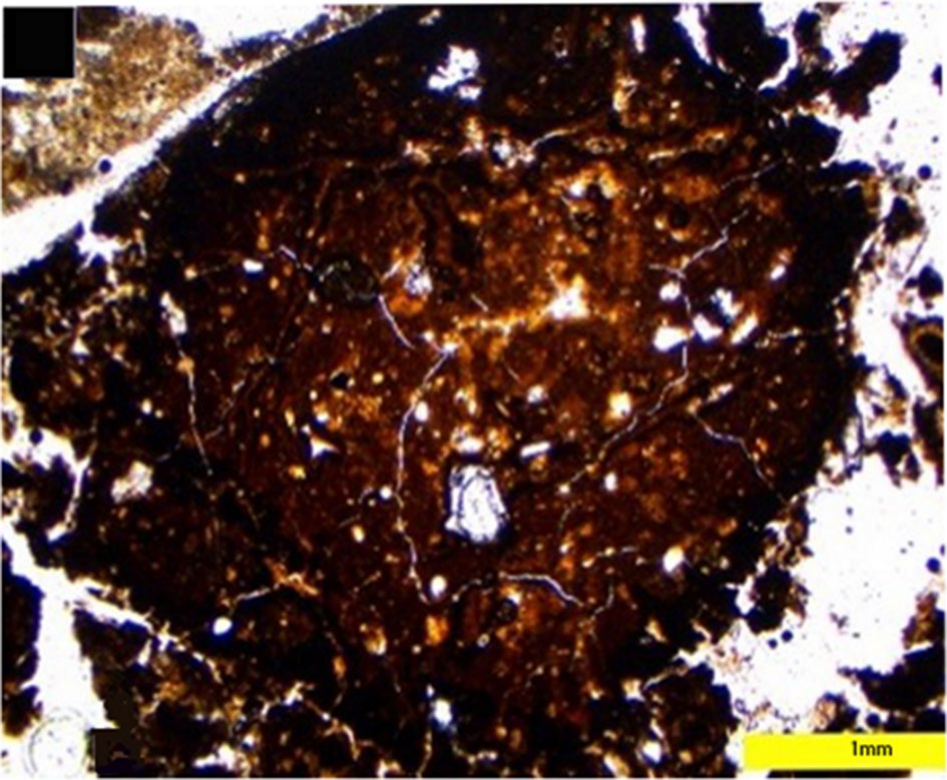
(**A**) Photomicrograph of a coprolite fragment containing course fraction and bone fragments in PPL from Thin section LA18-44 in Fig. 7, yellow bar denotes 1 mm in size.

The distinguishing features of carnivorous coprolites in thin section are size, morphology and a phosphatic composition containing skeletal and hair inclusions (e.g. Chin et al.^41^; Shillito et al.^42^; Nicosia & Stoops^43^; Morley et al.^36^). The coprolites, possibly related to tigers—known to make use of caves as a den in which to raise young or a shelter in which to consume prey (e.g. Tate^44^; Schaller^45^; Baryschnikov^46^)—were excavated from Lida Ajer, for example, the specimens in Fig. 11A, have an amorphous groundmass and phosphatic matrix similar to specimens from carnivorous animals including hyena and wolf (e.g. Fig. 4C & D in Morley et al.^36^), lynx, mountain lion, jaguar and human (e.g. Figs. 7.11A, 7.12A, 7.13A 7.14A in Brönnimann et al.^47^). The potential tiger coprolite has a significantly denser and compacted, slightly darker outer zone up to 1 cm thick (Fig. 11A) – a feature also commonly seen in hyena excrement. Coarse faction is a common component of carnivorous excrement; embedded silt and sand grains are evident in potential tiger coprolites (Fig. 11A) and hyena excrement (Fig. 4C in Morley et al.^36^), fine sand‐sized quartz grains are evident in lynx excrement (Fig. 7.11A in Morley et al.^36^), abundant subrounded mineral grain fraction, mostly comprised of sandy quartz, and rounded quartz grains are visible in the outer cortex of jaguar excrement (Fig. 7.13A in Brönnimann et al.^47^). Another common inclusion in omnivorous and carnivorous excrement specimens in thin section are centimetre-size bone fragments (e.g. Rodríguez et al.^48^; Brönnimann et al.^47^; Morley et al.^36^). Many bone fragments in the Lida Ajer coprolite thin sections show rounded edges due to digestion processes (e.g. Fig. 11A), though the bone in the lynx specimens are subangular (Fig. 7.11A in Morley et al.^36^). The thin section samples also have decomposed amorphous plant tissues – sometimes with preserved phytoliths—and pseudomorphic voids after decomposed hair, fur or wool (Fig. 11) (Horwitz & Goldberg^49^; Rodríguez et al.^48^; Macphail & Goldberg^50^; Sanz et al.^51^). There are some irregular scat morphologies and diagenetic alterations that could be potentially associated with a range of preservation states, e.g. post-depositional compression. Mustelids, viverrids, felids, and varanids are the amongst the predatory carnivores in the Lida Ajer and Ngalau Gupin faunal assemblages (De Vos^28^; Smith et al.^10^). Furthermore, a recent taxonomic and taphonomic survey of the faunal remains in Ngalau Gupin by Smith et al.^10^ have suggested that the ungulate and primate age profiles are most consistent with the hunting behaviour of a large carnivore, such as a tiger.

It is, however, difficult to establish if there are two or more producers from thin section analyses and other carnivore(s) may have contributed to these deposits. The ubiquitous accumulations of coprolites throughout several discrete sedimentary units indicates that the carnivores used the sampled area of the cave throughout the time represented by the preserved sediments. This evidence suggests the cave was used intermittently as a denning site. We propose the potential value behind further coprolite genomic analysis at Lida Ajer cave. Morphological inspection of coprolites in Lida Ajer cave has described the size, shape, colour, and inclusions evident on the deposit’s surface. As morphological analyses are often not diagnostic, further studies should be undertaken to determine the chemical and physical composition and observe greater palaeobiological data. Previous taphonomic research has revealed coprolites as a multi-proxy source of health, diet, archaeological and palaeontological contextual data (e.g. Chin et al.^51^; Backwell et al.^52^; Shin et al.^53^; Reinhard et al.^54^; Shillito et al.^55^). Analysing the composition and integrity of skeletal inclusions in carnivore coprolites can reveal digestive processes of the producer, prey selection patterns and taxonomic identification of small fauna in the local paleoenvironment (e.g. Erikkson et al.^56^; Bajdek et al.^57^; Dentzen-Dias et al.^58^; Barrios de Pedro et al.^59^).

### Reworking in Lida Ajer & Ngalau Gupin caves microstratigraphic record

The fossil assemblages of Lida Ajer and Ngalau Gupin are predominantly comprised of bone fragments and isolated teeth. A large proportion of the isolated teeth show chisel marks on the roots, which are typical of porcupine gnawing (e.g. Lenoble et al.^60^; Bacon et al.^61^; Bacon et al.^62^; Zeitoun et al.^63^). There are also porcupine remains found in Lida Ajer and Ngalau Gupin caves (de Vos^28^; Smith et al.^10^). Significant disturbance of the sediments by bioturbation can be seen in thin section, particularly at Ngalau Gupin. In particular, the sediments from the lower sample section of the deposit have been subject to extensive reworking. There are numerous infilled burrows throughout the sediment at the scale of 200 to 500 μm, so it is likely the sediment has also been reworked by substantial insect activity and percolation. Bioturbation and diagenesis are common and aggressive degradation processes acting upon cave sediments in Southeast Asia as increased temperatures and humidity in the tropics accelerate the pace of diagenetic change in the burial environment (Karkanas et al.^64^; Mijares & Lewis^65^; Morley & Goldberg^9^; Morley et al.^16^; Stephens et al.^14^; McAdams et al.^66^).

There is no fossil evidence in the Ngalau Gupin micromorphology sample. This supports the hypothesis from Smith et al.^10^ that the fossil remains excavated from the site were redeposited from the consolidated breccia NG-A site and only deposited on top of a more recent bat and insect accumulated sedimentary bed.

In addition to the insect activity that may have disturbed the sediments, our results suggest rapid deposition has also acted as an important ‘mixing’ mechanism in the lower sedimentary section of Lida Ajer deposits. Neutron tomographic imaging of intact breccia samples from Lida Ajer by Smith et al.^11^ suggest the breccia formed by several rapid depositional phases of water and sediment gravity flow. Micromorphological analysis reaffirms the neutron imaging data, and results suggest deposition is controlled by intervals of high recurrence floods and low energy stream flow. Low flow conditions are marked by thin beds of dusty clays, silt and evaporites in the LA18-41 samples. Transfer to a high-energy regime reworked sediment from external sources, marked by a transition from these thin beds to a heterogeneous array of allogenic clasts and chaotic matrix. Peak flow is marked by increases in deposition of speleothem and a transition to clay-rich sediments in the LA-43 samples. Episodic colluviation and slope wash dumped dense accumulations of amorphous sediments and triggered slumping events, leading to the partially fractured crusts of incorporated clast fragments and parallel striated pattern to the clay particles in the matrix of the LA18-44 samples. This preferential orientation of clay aggregates creates a halo of interference colours around the grains in cross-polarised light. Wetting and drying cycles of these unconsolidated clay-rich sediments then created shrink-swell episodes, generating differential sediment settlement that broke up the deposits (e.g. Kong & Tan^67^; Vogel et al.^68^).

Smith et al.^10^ hypothesises that the unconsolidated sediments of Ngalau Gupin are likely to have been reworked from the overhanging consolidated fossiliferous breccia of the site. The shrink-swell episodes and breaking up of the fossiliferous deposits seen in thin section may well prove the mechanism by which the reworking occurs.

### Diagenesis in Lida Ajer & Ngalau Gupin caves microstratigraphic record

In tropical caves, the fossiliferous deposits are often exposed to significant deterioration caused by chemical and physical degradation brought about by the high temperatures and precipitation of the humid tropical region (Mayer et al.^69^; De Sousa et al.^70^; McAdams et al.^66^). In the sediment samples from both cave sites, we record intense chemical diagenesis that has altered the composition of the sediments. These chemical processes are major agents of destruction of the palaeontological record but in identifying and better understanding the rate and degree of diagenesis occurring in the Lida Ajer and Ngalau Gupin sediments, we can form a significant palaeoenvironmental interpretation.

The calcium carbonate-charged waters that saturated the Lida Ajer cave sediments precipitated neo-formed calcite in the matrix and infilled void spaces throughout the sediment sample. Hydrodynamic sorting in a high energy waterflow fragmented the remains, and the fossil remains incorporated into the micromorphological samples from Lida Ajer sinkhole taken from the larger whole assemblage were reduced to solely isolated skeletal fragments and a solitary tooth. The isolated bone fragments were heavily abraded by the circulating waters, which stained the surfaces to orange yellow tone due to the presence of manganese oxide and iron precipitated by loss of CO_2_, oxidisation, and evaporation (López-González^71^). Manganese staining is suggestive of a redoximorphic environment. There is no evidence of guano deposition in the sediments of Lida Ajer, and there is no evidence of bat occupation in the field or at the micro-scale.

There are hundreds of bats roosting in Ngalau Gupin, and thick layers of fresh bat guano are evident on the floors and walls. There are abundant features that are indicative of guano-driven diagenesis visible in the Ngalau Gupin thin-section samples and observed authigenic mineral suites indicate that sediments became acidic enough to break down the clays in the groundmass. Furthermore, there are abundant gypsum crystals, abundant chitin fragments, and decayed plant residues in the sediments that are associated with the presence of fresh bat guano (Karkanas & Goldberg^25^). Phosphate mineral precipitation is often related to the decomposition of guano and the development of acidic sedimentary environments (e.g. Shahack‐Gross et al.^26^; Stephens et al.^14^). The phosphatic alteration in the Ngalau Gupin sediments is significant overall, though this alteration is spatially discrete—numerous clasts still maintain original lithologies and, in NG19 (2) thin section, a high degree of water saturation in guano-laden sediments may have prevented the acidity surge required to alter the clasts at all. McAdams et al.^66^ shows that even without acidity, phosphate rich environments in association with decomposing guano may lead to severe clast alteration. The authigenic phosphate in the groundmass of the Ngalau Gupin sediment appears to be weathering to gypsum or an adjacent sulphate mineral. The mealy coloured clays common in the ground mass clay appears to reflect two or more stages of mineral authigenesis. This high degree of authigenesis is indicative of intense diagenesis, and this process may have progressed since an earlier phase of deposition. The organic remains in the sediments then decomposed and were replaced by iron and manganese oxide, which appears as black staining in the sediment groundmass.

Data from Smith^34^ suggests that the mammal remains in Lida Ajer were initially deposited in the landscape surrounding the cave, perhaps as the result of carnivore predation. The resulting death assemblages may have been subsequently scavenged by porcupines in the cave. Following this, neutron tomographic imaging of consolidated breccia samples from Lida Ajer by Smith et al.^10^ suggests that the basic mechanism of deposition for the incorporated vertebrate remains is limited to localised short-distance water transport or sediment gravity flow over a relatively short timescale. Smith et al.^11^ determine the primary agents responsible for clastic deposition in Lida Ajer are likely several rapid pulses of hydrogeological activity and sheetwash colluvial sedimentation, suggesting that the breccia sites were formed from host limestone breakdown products that were transported over short distances prior to redeposition. Therefore, it is likely that the fossils were deposited in close proximity to the cave or within the vicinity of the cave entrance and redeposited via several methods to a deeper chamber within the cave.

## Conclusion

It is unlikely that any one methodological approach could hope to completely elucidate the complex depositional and taphonomic histories of caves such as Lida Ajer and Ngalau Gupin. This is particularly true when considering tropical cave environments, where destructive processes related to the loss of sedimentary and stratigraphic features are exacerbated by the wet, humid climate. These destructive forces acting upon cave sediments are a natural record of the setting in which the depositional processes originated and the dynamics of environmental change in the ancient tropics. Thus, a thorough analysis of tropical cave sediments can provide a useful record of the natural controls of fossil deposition, alteration, and destruction. Micromorphological analyses of the isolated sedimentary exposures in Ngalau Gupin and Lida Ajer have proven a remarkable technique with which to extend our knowledge of the depositional and post-depositional history of caves in west Sumatra, and of taphonomic processes acting upon the faunal remains within. Analysing the sedimentary facies and diagenetic trends in both cave deposits has allowed us to form a detailed reconstruction of sediment transport, bioturbation, carnivore occupation and guano‐driven diagenetic change. Despite these samples representing only a small sub-sample of the extensive sediments preserved in Ngalau Gupin and Lida Ajer, our robust reconstruction of complex sedimentary cave evolution provides clear insights into the environmental background across an important transition in human history in Southeast Asia.

## Data Availability

All data generated or analysed during this study are included in this published article.
